# Albendazole Exhibits Anti-Neoplastic Actions against Gastric Cancer Cells by Affecting STAT3 and STAT5 Activation by Pleiotropic Mechanism(s)

**DOI:** 10.3390/biomedicines9040362

**Published:** 2021-03-31

**Authors:** Min Hee Yang, In Jin Ha, Jae-Young Um, Kwang Seok Ahn

**Affiliations:** 1 KHU-KIST Department of Converging Science and Technology, Kyung Hee University, Seoul 02447, Korea; didmini@naver.com; 2 Department of Science in Korean Medicine, Kyung Hee University, 24 Kyungheedae-ro, Dongdaemun-gu, Seoul 02447, Korea; jyum@khu.ac.kr; 3 Korean Medicine Clinical Trial Center (K-CTC), Korean Medicine Hospital, Kyung Hee University, Seoul 02447, Korea; ijha0@naver.com

**Keywords:** albendazole, STAT3/5, SHP-1, gastric cancer, ROS, apoptosis

## Abstract

Albendazole (ABZ) has been reported to display anti-tumoral actions against various maliganncies, but possible impact of ABZ on gastric cancer has not been deciphered. As aberrant phosphorylation of STAT3 and STAT5 proteins can regulate the growth and progression of gastric cancer, we postulated that ABZ may interrupt the activation of these oncogenic transcription factors. We found that ABZ exposure abrogated STAT3/5 activation, inhibited phosphorylation of Janus-activated kinases 1/2 and Src and enhanced the levels of SHP-1 protein. Silencing of SHP-1 gene by small interfering RNA (siRNA) reversed the ABZ-promoted attenuation of STAT3 as well as STAT5 activation and cellular apoptosis. In addition, these effects were noted to be driven by an augmented levels of reactive oxygen species caused by drug-induced GSH/GSSG imbalance. Thus, the data indicates that ABZ can modulate the activation of STAT3 and STAT5 by pleiotropic mechanisms in gastric cancer cells.

## 1. Introduction

Signal transducers and activators of transcription (STAT) family consists of seven diverse members, named STAT1 to STAT6, STAT5a, and STAT5b [1,2]. Accumulated evidences have indicated that STAT members can exhibit diverse actions under both physiological and pathological conditions [3,4,5,6,7]. Of the STAT family members, STAT3 and STAT5 can regulate various hallmarks that are closely associated with tumorigenesis [8,9,10,11,12,13]. Phosphorylation of STATs can be driven via the stimulation of Janus-activated kinases (JAK) and Src families of proteins [14,15,16]. The activation can promote their homodimerization, movement into the nucleus, DNA binding, thus leading to transcription of oncogenic genes [4,17,18]. Interestingly, protein tyrosine phosphatases (PTPs) such as SHP-1, SHP-2, PTPε, and PTEN can negatively regulate the STAT signaling pathway [19,20,21]. In addition, various previous reports have indicated that a number of naturally derived pharmacological agents can attenuate STAT activation, which may have a great potential in the prevention and therapy of cancer [13,22,23,24,25,26].

Gastric cancer remains a lethal disease [25,27,28,29]. It has emerged as a significant health problem characterized by poor prognosis and disease relapse [30,31,32]. Gastric cancer has been treated using different strategies such as surgery, chemotherapy, and radiation therapy, but the clinical outcome has been not quite effective with development chemoresistance being a major obstacle [31,33,34,35,36,37]. Hence, elucidation of the biological characteristics and molecular mechanisms of novel agents may be beneficial for the gastric cancer treatment. As the abnormal expression and dysregulation of STAT3/5 have been reported in various malignancies including gastric cancer [38], targeting of STAT3/5 phosphorylation may be beneficial for gastric cancer treatment.

A number of agents isolated from medicinal plants have been used for treatment against diverse malignancies [39,40,41,42]. Albendazole (Methyl 5-(propylthio)-2-benzimidazolecarbonate)) carbamate, ABZ), a well-known benzimidazole derivative carbamate anthelminthic [43], can target helminth cell proliferation through disputing microtubule assembly and affecting glucose uptake [44,45]. ABZ has been reported to exhibit diverse anticancer actions against several tumor cell lines such as hepatocellular carcinoma, colorectal cancer, non-small cell lung cancer, as well as cutaneous squamous cell carcinoma [46,47,48,49]. For instance, ABZ was reported to be effective against non-small cell lung cancer cells by reducing both vascular endothelial growth factor (VEGF and hypoxia-inducible factor-1-α (HIF-1-α) activities [48]. It was also found to promote apoptosis in human leukemia cells by causing TNF-α upregulation [50]. However, the influence of ABZ on gastric cancer cells and the underlying mechanisms have not been evaluated previously. In this study, it was investigated whether ABZ can impact the activation of STAT3 and STAT5 pathway in gastric cancer cells and thereby exhibit pleiotropic actions on tumor progression as well as survival.

## 2. Materials and Methods

### 2.1. Reagents

Albendazole (ABZ), 3-(4,5-dimethylthiazol-2-yl)-2,5-diphenyltetrazolium bromide (MTT), Tris base, glycine, NaCl, sodium dodecylsulfate (SDS), and bovine serum albumin (BSA) were purchased from Sigma-Aldrich (St. Louis, MO, USA). LightShift^®^ Chemiluminescent EMSA kit and Trizol were purchased from Thermo Fisher Scientific Inc. (Waltham, MA, USA). FITC Annexin V Apoptosis Detection Kit was purchased from BD Pharmingen™ (BD Biosciences, Becton-Dickinson, Franklin Lakes, NJ, USA). TUNEL (terminal transferase mediated dUTP-fluorescein nick end labeling) assay kit was from Roche Diagnostics GmbH (Mannheim, Germany). Anti-p-STAT3(Tyr705), anti-p-STAT5 (Tyr694/Tyr699), anti-p-JAK1 (Tyr1022/1023), anti-JAK1, anti-p-JAK2 (Tyr1007/1008), anti-JAK2, anti-p-Src (Tyr416), anti-Src, anti-Cleaved caspase3, and anti-Cyclin D1 antibodies were purchased from Cell Signaling Technology (Beverly, MA, USA). Anti-STAT3, anti-PTPε, anti-SHP-1, anti-Procaspase-3, anti-PARP, anti-Bcl-2, anti-Bcl-xL, anti-Survivin, anti-Cyclin D1, anti-COX-2, anti-β-actin antibodies, and SHP-1 siRNA were purchased from Santa Cruz Biotechnology (Santa Cruz, CA, USA).

### 2.2. Cell Lines and Culture Conditions

SNU-1 and SNU-16 cells were obtained from the Korean Cell Line Bank (Seoul, Korea). Human normal gastric epithelia mucosa GES-1 cells were provided by Dr. Sang-kil Lee (Division of Gastroenterology, Department of Internal Medicine, Institute of Gastroenterology, Yonsei University College of Medicine, Seoul, Korea). SNU-1, SNU-16, and GES-1 cells were cultured in RPMI-1640 medium containing 10% FBS.

### 2.3. MTT Assay

SNU-1, SNU-16, and GES-1 cells were treated with ABZ (0, 10, 30, 50, 100 µM) for 24 h. The cell viability was measured to select the most suitable cell lines for analyzing the anticancer potential of ABZ. After 24 h of treatment, 30 µL of MTT solution (2 mg/mL) for 2 h, then MTT lysis for overnight. The cell viability was analyzed with absorbance of MTT formazans using by VARIOSKAN LUX (Thermo Fisher Scientific Inc, Waltham, MA, USA) at 570 nm [51].

### 2.4. Western Blot Analysis

After the cells were exposed to varying concentrations and time intervals of ABZ, then the cells were harvested and lysed with lysis buffer (20 mM Tris (pH 7.4), 250 mM NaCl, 2 mM EDTA (pH 8.0), 0.1% Triton X-100, 0.01mg/mL aprotinin, 0.005 mg/mL leupeptin, 0.4 mM phenyl methane sulfonyl fluoride (PMSF), and 4 mM NaVO_4_) and the total protein concentrations were determined by Bradford reagent (Bio-Rad, Hercules, CA, USA). Equal amount of lysates resolved in a 10–15% SDS-polyacrylamide gel. After SDS-PAGE, the proteins were transferred onto nitrocellulose membrane, blocked with 5% skimmed milk in 1× TBST (1× TBST with 0.1% Tween 20) and proved with specific primary antibodies: anti-p-STAT3 (Tyr705), anti-p-STAT5 (Tyr694/Tyr699), anti-p-JAK1 (Tyr1022/1023), anti-JAK1, anti-p-JAK2 (Tyr1007/1008), anti-JAK2, anti-p-Src (Tyr416), anti-Src, anti-Cleaved caspase3, and anti-Cyclin D1, anti-STAT3, anti-PTPε, anti-SHP-1, anti-Procaspase-3, anti-PARP, anti-Bcl-2, anti-Bcl-xL, anti-Survivin, anti-Cyclin D1, anti-COX-2, and anti-β-actin antibodies. Antibodies were incubated at 4 °C for overnight. Finally, the membranes were incubated with horseradish peroxidase (HRP) conjugated anti-rabbit IgG antibodies, and anti-mouse IgG antibodies (diluted 1/5000 in TBST) at room temperature for 2 h. The membranes were detected using a chemiluminescence (ECL) (EZ-Western Lumi Femto, DOGEN, Seoul, Korea). After detection, the same blots were stripped for 1 h and reprobed with anti-β-actin antibodies to demonstrate equal protein loading [52].

### 2.5. EMSA

To determine whether ABZ can suppress the DNA binding capability of STAT proteins, electrophoretic mobility shift assay (EMSA) was performed. The nuclear extracts were prepared from ABZ-treated cells using 10× binding buffer, poly (di-dc), NP-40, and probe: 5′-biotinylated STAT3 oligonucleotide (5′-GATCCTTCTGGGAATTCCTAGATC-3′ and 5′-GATCTAGGAATTCCCAGAAGGATC-3′; BIONEER, Daejeon, Korea). STAT5 oligonucleotide (5′-AGA TTT CTA GGA ATT CAA TCC-3′ and 5′-GGA TTG AAT TCC TAG AAA TCT-3′). Oct-1 (5′-TTCTAGTGATTTGCATTCGACA-3′ and 5′-TGTCGAATGCA AATCACTAGAA-3′; BIONEER) was used for loading control. Protein-oligonucleotide complex was loaded on polyacrylamide gel and transferred to nylon membrane. Then cross-linked by 540 nm UV, blocked with blocking solution for 30 min and incubated with substrate solution for 5 min. Finally, the membrane was detected by using LightShift^®^ Chemiluminescent EMSA kit (Thermo Fisher Scientific) [8].

### 2.6. Immunocytochemistry

After SNU-1 and SNU-16 cells were treated with ABZ 50 µM for 3 h, the cells were fixed with 4% paraformaldehyde (PFA) at room temperature for 20 min and washed three times by 1× PBS. The cells were permeablized with 0.2% Triton-X 100 for 10 min and blocked using 5% BSA in PBS for 1 h. After that, the cells were incubated with anti-p-STAT3 (Tyr705), anti-p-STAT5 (Tyr694/Tyr699), anti-STAT3, and anti-STAT5 (1:100) for overnight at 4 °C Next day, cell were washed three times by 1× PBS and incubated with Alexa Fluor® 488 donkey anti-mouse IgG (H+L) antibody and with Alexa Fluor® 594 donkey anti-rabbit IgG (H + L) secondary antibodies (1:1000) at room temperature for 1 h. Then, stained with DAPI (1 µg/mL) for 3 min at room temperature and mounted in Fluorescent Mounting Medium (Golden Bridge International Labs, Mukilteo, WA, USA). Finally, the fluorescence signal was detected by using a FluoView FV1000 confocal microscope (Olympus, Tokyo, Japan) [53].

### 2.7. Transfection with SHP-1 siRNA

To knock-down the expression of SHP-1, SNU-1 and SNU-16 cells were transfected with SHP-1 and scrambled siRNA (100 nM) for 24 h using by Neon™ Transfection System (Invitrogen, Carlsbad, CA, USA). After transfection, cells were treated with ABZ for 3 h or 24 h.

### 2.8. Reverse Transcription Polymerase Chain Reaction (RT-PCR)

The cells were treated with ABZ (0, 10, 30, 50 µM) for 3 h or 50 µM of ABZ for (0, 12, 24, 36, 48 h). Total RNA was extracted with Trizol reagent. RNA was purified with chloroform and isopropanol. RNA was converted to cDNA using superscript reverse transcriptase and Taq polymerase by reverse transcription polymerase chain reaction (RT-PCR) (TAKARA, Tokyo, Japan). The relative expressions of SHP-1, Bcl-2, Bcl-xL, Survivin, MMP-9 were analyzed using PCR with glyceraldehyde-3-phosphate dehydrogenase (GAPDH) was used as an international control. Then PCR products were run on 1% agarose gel and stained with Loading Star (Dynebio, Seongnam, Korea). Stained bands were detected with UV light [54].

### 2.9. GSH/GSSG Assay

SNU-1 and SNU-16 cells were treated with ABZ 50 µM for 12 h and GSH/GSSG ratio was evaluated by GSH/GSSG-Glo Assay (Promega, Madison, WI, USA according to the manufacturer’s protocol [8].

### 2.10. Cell Cycle Analysis

To determine the effects of ABZ on cell cycle progression, cell cycle analysis was performed using propidium iodide. SNU-1 and SNU-16 cells were treated with ABZ 50 µM for 24 h. After that, cells were harvested and fixed by EtOH for overnight. Then, cells were resuspended in 1× PBS containing RNase A for 1 h at 37 °C and stained with propidium iodide. Cells were analyzed by BD Accuri™ C6 Plus Flow Cytometer (BD Biosciences, Becton-Dickinson) equipped with the BD Accuri C6 Plus software [55].

### 2.11. Annexin V Assay

The cells were treated with ABZ 50 µM for 24 h and Annexin V assay was done using Annexin V Apoptosis Detection Kit (BD Biosciences). After treatment, the cells were stained with FITC tagged Annexin V antibody and propidium iodide for 15 min at room temperature. Then stained samples were resuspended in 1× binding buffer, analyzed by BD Accuri™ C6 Plus Flow Cytometer (BD Biosciences) [56].

### 2.12. TUNEL Assay

Late apoptotic cell death was determined using a Roche Diagnosis TUNEL assay kit. SNU-1 and SNU-16 cells were treated with ABZ 50 µM for 24 h and fixed with 4% paraformaldehyde for 30 min and incubation with 0.2% triton X-100 for 10 min in fresh 1× PBS. Cells were washed by 1× PBS and incubated with TUNEL enzyme and TUNEL label for 1 h at 37 °C. Then cells were analyzed by BD Accuri™ C6 Plus Flow Cytometer (BD Biosciences) [57].

### 2.13. Statistical Analysis

All the numerical values have been represented as the mean ± SE. Statistical significance of the data was deciphered by Mann-Whitney U test. Significance was set at *p* < 0.05.

## 3. Results

### 3.1. ABZ Suppresses the Cell Viability

**Figure 1:**
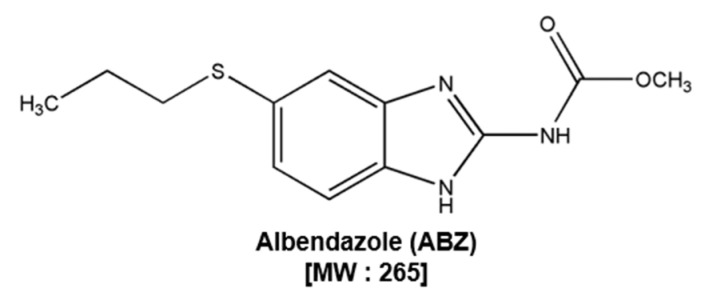
The structure of albendazole (ABZ).

**Figure 2:**
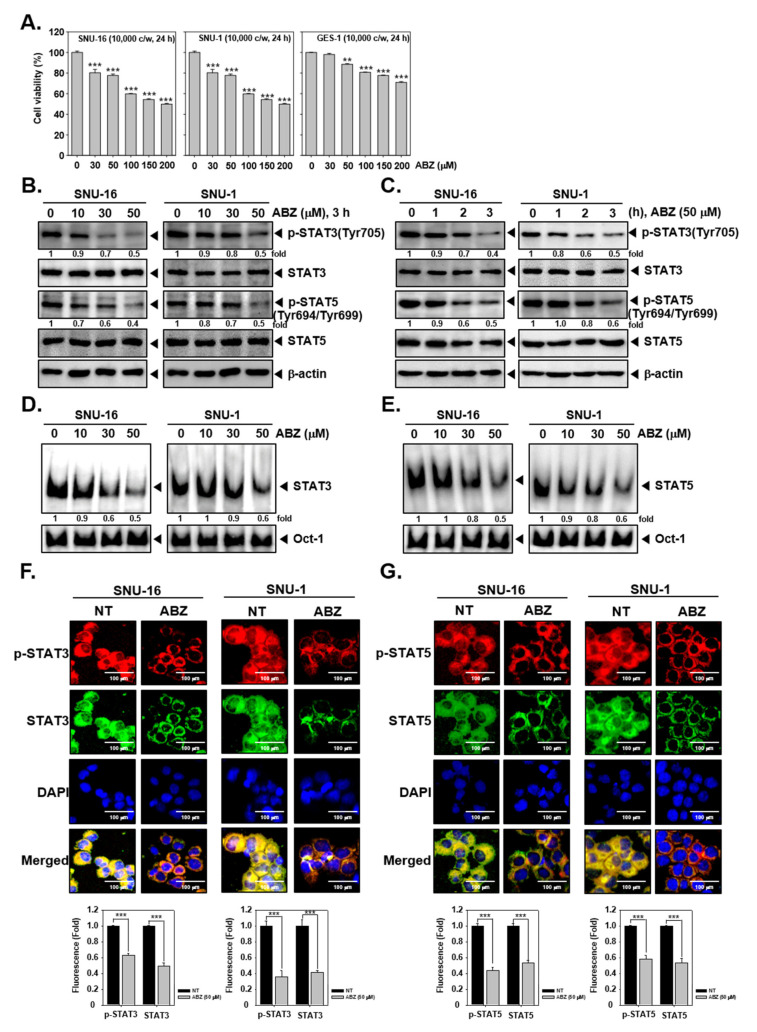
ABZ affects STAT3/5 phosphorylation. (**A**) SNU-16, SNU-1, and GES-1 cells were treated with ABZ (0, 10, 30, 50, 100 µM) for 24 h, then viability deciphered by MTT assay. (**B**) SNU-16 and SNU-1 cells (1 × 10^6^ cells/well) were treated with ABZ (0, 10, 30, 50 µM) for 3 h and western blotting was done. (**C**) Cells were treated with 50 µM of ABZ for (0, 1, 2, 3 h) and western blotting was carried out (**D**,**E**) SNU-16 and SNU-1 cells were treated as described above in panel C and nuclear STAT3 and STAT3 levels were determined. (**F**,**G**) The cells were treated with 50 µM of ABZ for 3 h and intracellular p-STAT3 and p-STAT5 distribution was evaluated by immunocytochemistry. Data represent means ± SD. ** *p* < 0.01 vs. non-treated (NT) cells, *** *p* < 0.001 vs. non-treated (NT) cells. The results shown are representative of three independent experiments.

The structure of ABZ is shown in Figure 1. The cytotoxic actions of ABZ against SNU-16 and SNU-1 and normal gastric GES-1 cells was evaluated by MTT assay. We observed that ABZ displayed higher cytotoxic effects against SNU-16 and SNU-1 as compared to GES-1 cells, thereby suggesting its selectivity against tumor cells (Figure 2A).

### 3.2. ABZ Substantially Affected STAT3 and STAT5 Phosphorylation

In order to identify if ABZ can alter STAT3/5 activity, SNU-16 and SNU-1 cells were treated with different doses of drug for 3 h or exposed to different time periods at 50 µM ABZ concentration. As shown in Figure 2B,C, constitutive STAT3 and STAT5 phosphorylation was substantially suppressed upon ABZ exposure. Moreover, as depicted in Figure 2D,E, ABZ could markedly attenuate the DNA-binding activities of STAT proteins. Additionally, as shown in Figure 2F,G, ABZ also effectively reduced the nuclear translocation of STAT3 and STAT5 proteins thus preventing the gene transcription.

### 3.3. ABZ Alters Phosphorylation of JAK1/2 and Src Kinases

**Figure 3:**
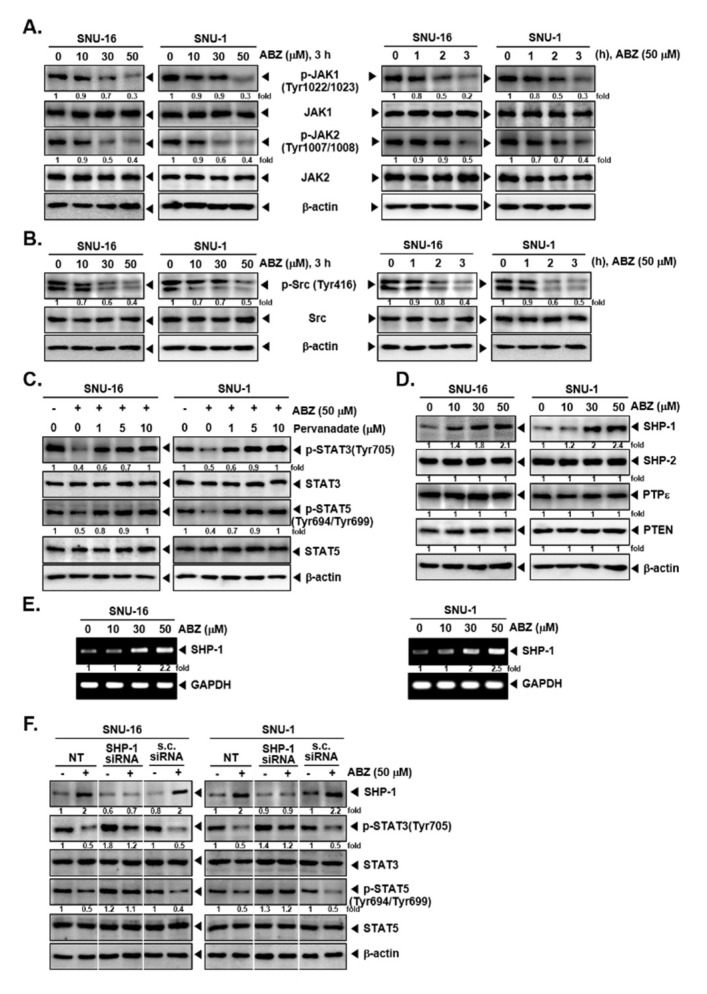
ABZ promotes protein SHP-1 levels in gastric carcinoma cells. (**A**,**B**) SNU-16 and SNU-1 cells were treated with (0, 10, 30, 50 µM ABZ) in a time dependent fashion (0, 1, 2, 3 h) and western blotting was performed. (**C**) The cells were pre-treated with various concentrations of pervanadate for 30 min, then incubated with 50 µM of ABZ for 3 h and western blotting was performed. (**D**) Cells were treated with 50 µM for 3 h, and western blot analysis was done. (**E**) RNA levels of SHP-1 was evaluated by RT-PCR. (**F**) Cells were transfected with scrambled or SHP-1 specific siRNA (100 nM). After 24 h, cells were treated with ABZ 50 µM for 3 h and the western blotting was done. The results shown are representative of independent experiments.

We next deciphered if ABZ can alter the levels of kinases involved in regulating activation of STATs. As shown in Figure 3A,B, ABZ substantially downregulated the activation of JAK1, JAK2 and Src kinases in a concentration and time-controlled fashion in SNU-16 and SNU-1 cells.

### 3.4. Tyrosine Phosphatases Affect STAT3/5 Modulatory Actions of ABZ

To analyze the mechanism of ABZ-stimulated inhibition of STATs phosphorylation, we examined the effect of ABZ in modulating the levels of protein tyrosine phosphatases (PTPs). It was noted that the exposure to sodium pervanadate reversed the attenuation of STAT3 and STAT5 phosphorylation, highlighting that this activity of ABZ can be regulated by a tyrosine phosphatase (Figure 3C). We next observed that ABZ only induced SHP-1 expression but did not affect other PTPs (SHP-2, PTPε, PTEN) in both SNU-16 and SNU-1 cells. ABZ also augmented the mRNA levels of SHP-1 (Figure 3E). Additionally, upon SHP-1 knockdown, SHP-1 expression was substantially reduced and ABZ was unable to affect STAT3 and STAT5 phosphorylation in SHP-1 knocked down cells (Figure 3F). 

### 3.5. ABZ Alters the Levels of Various Oncogenic Proteins and Caused Apoptosis

**Figure 4:**
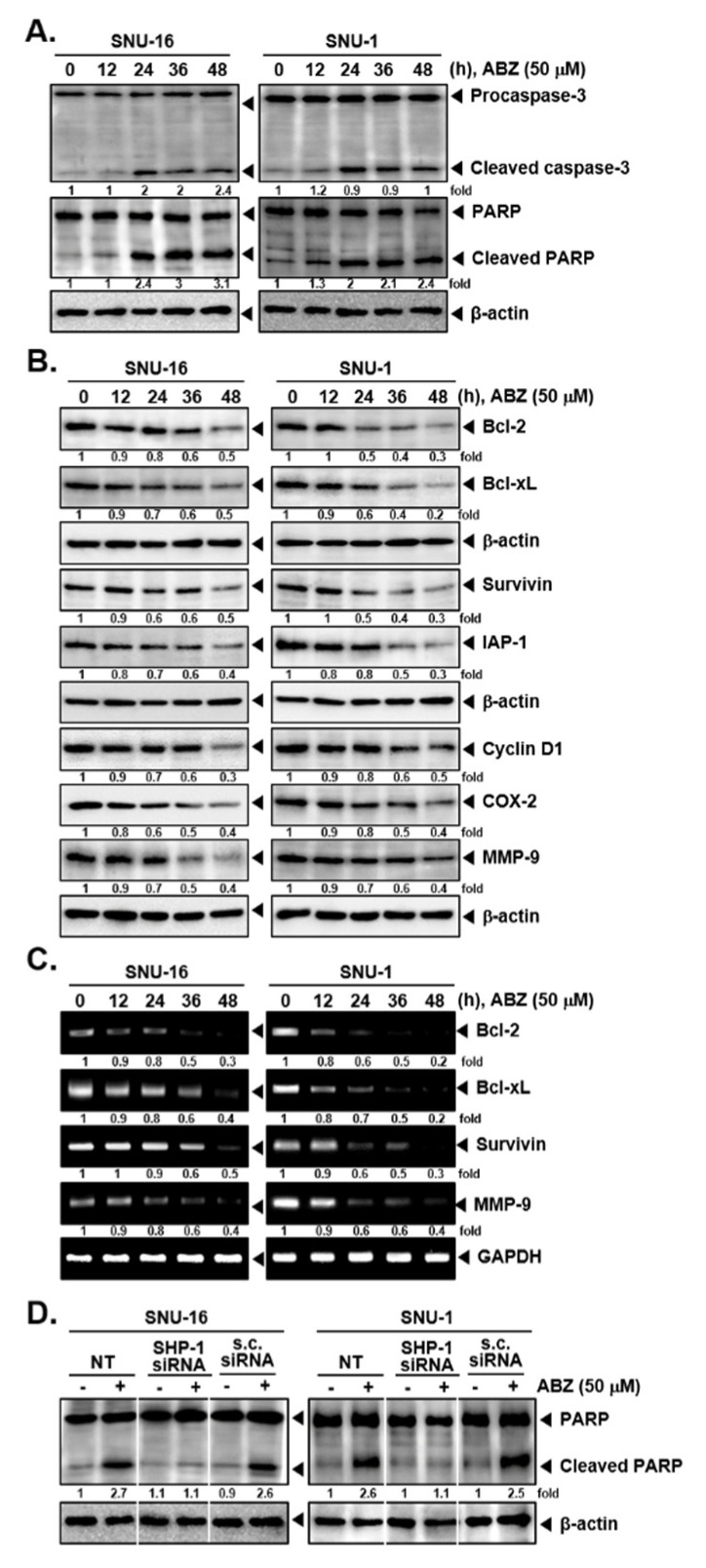
ABZ promotes activation of caspase-3 and PARP cleavage. (**A**,**B**) SNU-16 and SNU-1 cells were treated with ABZ 50 µM for (0, 12, 24, 36, 48 h) and levels of various proteins were examined by western blot analysis. (**C**) Cells were treated as described above in panel (**A**,**B**), total RNA was extracted and examined for expression of Bcl-2, Bcl-xL, Survivin, and MMP-9 by RT-PCR. (**D**) Cells were transfected as described in Figure 2F and the western blotting for PARP was performed.

SNU-16 and SNU-1 cells exposed to 50 µM of ABZ and western blotting for apoptotic markers was done. As shown in Figure 4A, ABZ promoted apoptosis by increasing the breakdown of caspase-3 and PARP proteins. ABZ also downregulated the expression of diverse proteins involved in regulating various hallmarks of tumor growth at protein and mRNA levels (Figure 4B,C). As shown in Figure 4D, the deletion of SHP-1 also attenuated ABZ-induced PARP cleavage which suggested that SHP-1 may play a vital role in regulating anti-cancer properties of ABZ.

### 3.6. ABZ Induces Apoptotic Cell Death in Gastric Cancer Cells

**Figure 5:**
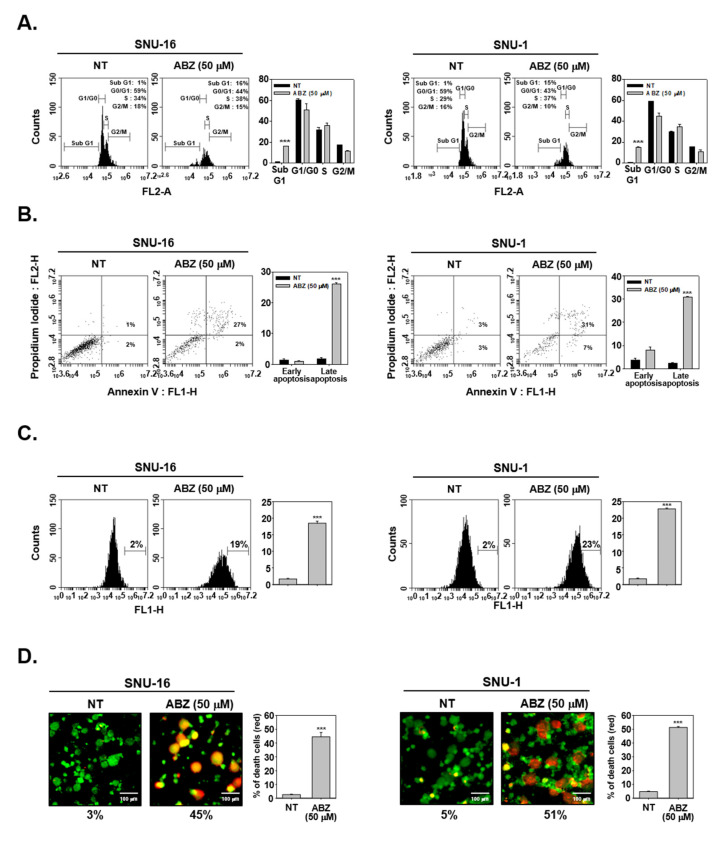
ABZ induces potential apoptosis. (**A**–**C**) SNU-16 and SNU-1 cells were treated with ABZ 50 µM for 24 h and apoptotic cells were analyzed through cell cycle analysis, Annexin V and TUNEL assays. (**D**) Cells were treated as described above in panel (**A**–**C**), Live and dead assay was done. Live cells were stained in green and dead cells were stained in red. The graph shows quantification of the population of dead cells. Data represent means ± SD. *** *p* < 0.001 vs. non-treated (NT) cells.

The impact of ABZ on apoptosis was studied using cell cycle analysis, annexin and TUNEL assays. Early apoptotic cells are annexin V-FITC+/PI-, whereas late apoptotic cells are annexin V-FITC+/PI+. As shown in Figure 5A, ABZ enhanced aggregation of cell population in sub G1 phase. And ABZ produced late apoptosis (annexin V-FITC+/PI+) as evidenced by shifting of peak to right side (Figure 5B,C). The results of live and dead assay also confirmed that ABZ also significantly attenuated viability of gastric cancer cells (Figure 5D).

### 3.7. ABZ Exhibits Anti-Neoplastic Effect through ROS-Mediated Events

**Figure 6:**
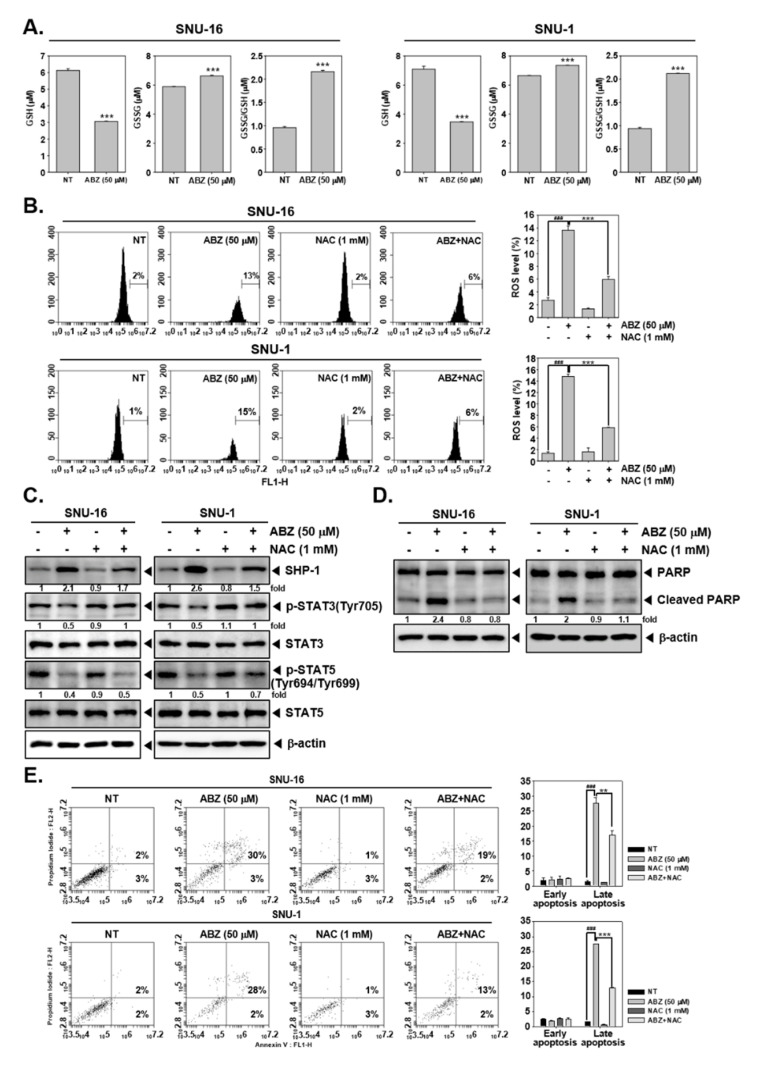
Regulation of ROS production by ABZ. (**A**) SNU-16 and SNU-1 cells were treated with ABZ 50 µM for 12 h and GSH/GSSG assay was carried out. (**B**) Cells were pre-treated with NAC 1mM for 15 min, then incubated with ABZ 50 µM for 12 h. and ROS production was measured (**C**) Cells were pre-treated with NAC 1 mM for 15 min, then incubated with ABZ 50 µM for 3 h. and western blotting was executed. (**D**) Cells were pre-treated with NAC 1 mM for 15 min, then incubated with ABZ 50 µM for 24 h. and western blotting was conducted. (**E**) Cells were treated as described above in panel (**D**), and Annexin V assay was performed. Data represent means ± SD. ^###^ *p* < 0.001 vs. non-treated (NT) cells, ** *p* < 0.01 vs. ABZ (50 μM) treated cells, *** *p* < 0.001 vs. ABZ (50 μM) treated cells.

To explore the potential involvement of ROS in observed anti-cancer functions of ABZ, we studied the impact of ABZ on the GSH/GSSG system. As demonstrated in Figure 6A, ABZ decreased GSH levels in SNU-16 and SNU-1 cells. On the contrary, GSSG and GSSG/SGH ratio was noted to be increased. Additionally, to validate if ABZ can generate oxidative stress, ROS levels were quantitated through H_2_DCF-DA staining. A significant increase in ROS levels were noted upon ABZ exposure and antioxidant NAC prevented ABZ-induced ROS production (Figure 6B). Interestingly, pretreatment of NAC only partially suppressed SHP-1 induction and abolished STAT3 (SNU-16 and SNU-1 cells) and STAT5 (SNU-16 cells) suppression caused by ABZ treatment (Figure 6C). Furthermore, antioxidant pretreatment could also substantially mitigate apoptosis promoted by ABZ, thereby demonstrating that oxidative stress can regulate pro-apoptotic actions of ABZ (Figure 6D,E). 

## 4. Discussions

**Figure 7:**
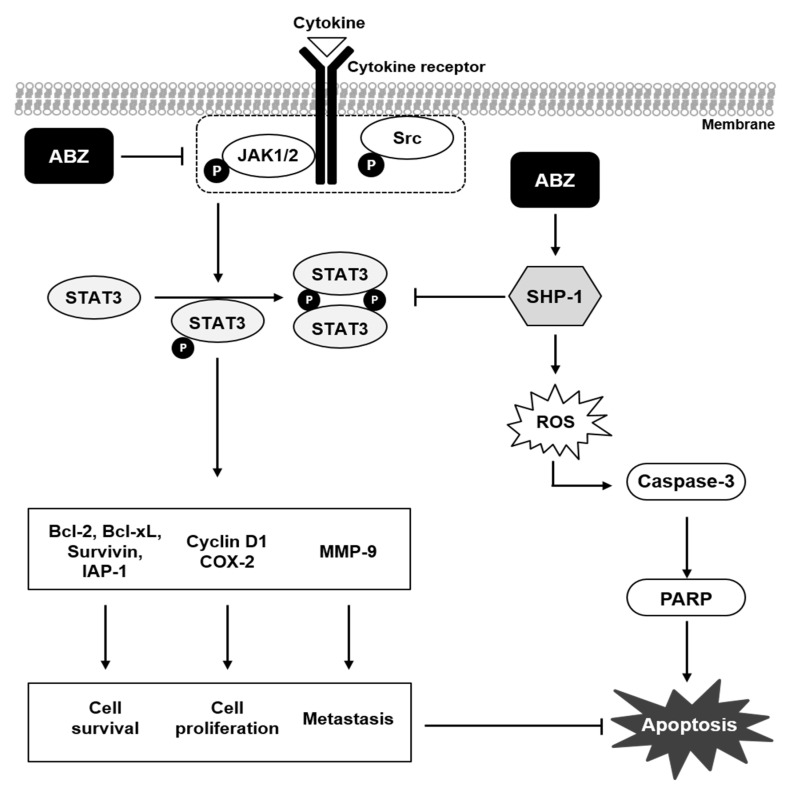
A schematic diagram showing the effects of ABZ on STAT3/5 signaling pathway via modulating SHP-1 expression.

Previous reports have indicated that ABZ may display significant anti-tumor activities in different tumor models, with gastric cancer being an exception [48,58]. The purpose here was to elucidate the anti-cancer impact of ABZ and also to unravel its mode of actions. We found that ABZ targeted both STAT3 and STAT5 activation, up-regulated the induction of SHP-1 protein and promoted ROS accumulation which can lead to substantial apoptosis (Figure 7).

STAT3 and STAT5 proteins acting as potential oncogenes have often reported to be overexpressed in different cancers [4,11,59]. A number of prior studies have found that pharmacological agents derived from natural sources can modulate the activation of different members of STAT family of proteins and attenuate tumorigenesis [8,10,13,24,60]. We found for the first time that ABZ could mitigate STAT3 (Tyr705 residue) as well as STAT5 (Tyr694/Tyr699 residue) activation and also negatively affect DNA-binding activity and nuclear translocation of these proteins. The phosphorylation of JAK kinase can lead to STAT3 and STAT5 activation in diverse tumor cell lines, including gastric carcinoma cells [15,61]. We noted that ABZ could substantially reduce phospho- JAK1, JAK2, and Src levels thus emphasizing that it may not be only affecting to STAT proteins.

A number of PTPs can regulate both STAT3 and STAT5 signaling, such as SHP-1, SHP-2, PTPε, etc [62,63,64]. We also found that ABZ-caused abrogation of STAT3 and STAT5 activation may be regulated by alteration in SHP-1 level. Indeed, we found that ABZ exposure augmented the levels of SHP-1 protein and mRNA levels, but the silencing of this phosphatase could neutralize the ABZ-observed impact on the phosphorylation of STAT proteins as well as on apoptosis. It has been suggested that various pharmacological agents may exert their anti-tumor effect through the up-regulation of SHP-1 expression [13,19,65,66]. Baek et al., found that ginkgolic acid 17:1 can induce substantial apoptosis via the up-regulation of SHP-1 [65]. Interestingly, ABZ was found to promote SHP-1 expression, thereby clearly indicating that the SHP-1 play an important role in the down-regulation of STAT3 and STAT5 by ABZ.

Haifeng et al., found that ABZ suppressed cell proliferation and induced late apoptosis in human pancreatic cancer cells [67]. We also noted that ABZ can mitigate the expression of various STAT3-regulated genes that could stimulate cell proliferation, prevent apoptosis and promote angiogenesis. These included proteins controlling anti-apoptosis (Bcl-2, Bcl-xL, Survivin, and IAP-1), cell cycle regulation (Cyclin D1), cell proliferation (COX-2), and angiogenesis (MMP-9 and VEGF). These observations corroborate with apoptosis induction as indicated by the increase of caspase-3-induced PARP cleavage, and an increase in the number of apoptotic cells as detected by cell cycle, annexin V and Live and Dead assays. Abnormal expression of STAT3 and STAT5 can function as pro-survival mechanism to enhance tumor survival as well as growth and hence targeting these proteins by agents such as ABZ could lead to apoptosis. 

ROS generally functions as a double-edge sword can function both as tumor promoter and suppressor under different conditions [68,69]. In general, low levels of ROS can regulate cell proliferation. However, increased ROS levels can cause damage to proteins, nucleic acids, lipids, membranes and organelles, which can lead to oxidative stress induced cell death processes such as apoptosis [70]. Moreover, our group has previously identified other STAT3 and STAT5 blockers namely formononetin and ophiopogonin D, which can exert significant anti-tumor effects through increased ROS production in different tumor models [8,71]. As a regulator of cell signaling, SHP-1 modulates the production of ROS and can negatively regulate STAT3 and STAT5 activation [72,73]. Interestingly, it was found that STAT3/5 abrogation and apoptosis triggered by ABZ could be negated by exogenous antioxidant, NAC. However, ABZ-induced SHP-1 was found to be only partially suppressed by NAC and this interesting observation may require additional investigation, although ABZ primarily appears to exert its effects by modulating SHP-1 expression. Overall our findings indicate that ROS could possibly regulate the pleiotropic anti-cancer actions of the drug and SHP-1 may be modulated by ROS production. Indeed ABZ exposure lead to an augmentation in GSSG/GSH ratio, which effectively served as a major source of ROS in driving the impact of drug on the tumor survival and proliferation. Overall, ABZ can serve as an efficient blocker of both STAT3 and STAT5 proteins by promoting oxidative stress as well as inducing expression of SHP-1 and thus exhibit its multifaceted actions against gastric cancer cells, which need to be validated in preclinical studies in future.

## Data Availability

The data presented in this study are available on request from the corresponding author.
